# Tackling structural inequalities to reduce concussion-related dementia in sportspeople

**DOI:** 10.18632/aging.204431

**Published:** 2022-12-07

**Authors:** Valentina Gallo, Damien McElvenny

**Affiliations:** 1 University of Groningen, Campus Fryslân, Leeuwarden, The Netherlands

**Keywords:** concussion, cognitive decline, rugby, sport, structural inequalities

In contrast with the neuropathological evidence accumulating on Chronic Traumatic Encephalopathy (CTE) [1] and its relation to concussion and the severe clinical consequences, the evidence coming from the sport research is not always consistent. For example, a recent study conducted among former elite English rugby players, the BRAIN study, found no overall evidence of an association between number of concussions suffered when playing and cognitive function at an older age [2]. The study, featuring an in depth evaluation of cognitive function, found an increased risk of poorer cognition in those who suffered three or more concussions compared to none, but only after age 70-75 years. This suggests the presence of an association which is delayed compared to that found in previous studies conducted among French, Scottish, and New Zealand rugby players [3]. If the effect in English rugby players is a true effect, why might such a delayed effect on cognition occur when compared to other rugby players, or players of other contact sports?

It is possible that the reason needs to be researched in the characteristics of this same group of sportspersons, that is, with very high socio-economic status. In England, rugby union is a sport played by players from a higher than average socioeconomic status. Among the English rugby players involved in the BRAIN study, three out of four have at least an undergraduate university degree, with one in three holding a postgraduate degree. Three quarters of the former players were working as legislators, senior officials, managers or professionals. Conversely, 52% of the French rugby players and between 18% and 26% of the New Zealand had a high school degree or above; Scottish players had a mean number of years spent in education of 16 (SD 6.2) [3].

Epidemiological studies have suggested that some individual cognitive circumstances such as education attainment, occupational demand, and type of leisure activities can increase cognitive reserve. This in turn can provide an increased resilience of the brain to neuropathological changes delaying the detrimental effect of external exposures, which might explain the difference in age onset of the symptoms between the BRAIN study and the other rugby studies [4]. The higher the reserve the greater is the capacity of people to compensate small deficits in cognition, and the more delayed their clinical relevance. It is reasonable to assume that the population of English rugby players included in the study by Gallo et al. [2], for the characteristics described above, display overall a higher cognitive reserve than their international counterparts.

Recently, further evidence was provided on the process of embodiment of social factors, with a specific focus on social stress and inequalities, through the study of allostatic load. Allostatic load is a way to capture embodiment of social stress measuring the global physiological ‘wear and tear’ which results into the adaptation to the social environment to the stress response system over the lifespan. This allows the direct measurement of the effects of social stress such as structural discrimination, poverty, and racism in blood biomarkers [5]. Interestingly, social stress measured through allostatic load is associated with a cognitive and physical decline [6]. Importantly, it is unlikely that a physical damage as concussion could alter the stress reaction measured by allostatic load; no differences in allostatic load across concussion groups was found among rugby players in Scotland [7].

The ethnically uniform, highly educated, and overall high socio-economic status of the sample of English rugby players from the BRAIN study is likely to be exposed to less social stress compared to their international counterparts, and other contact players whose tradition is less linked to varsity sport in prestigious universities. These mechanisms of social protection are likely to explain also the late, if any, onset of cognitive impairment associated with youth concussion. If confirmed by further studies, this would reinforce the imminent need to close the inequality gap between and within populations in order to protect individual health from the embodiment of eternal exposures, including concussions.

Education and reducing structural inequalities might be the best option we have in public health to reduce the burden of cognitive decline. This is particularly relevant for those populations experiencing an inexorable ageing process, many of which are in low-resource settings. In these populations, delaying clinical onset of diseases at population level is a key component of a population-based preventive approach, one of the key goals of the recently established International Research Network on Dementia Prevention [8].
